# Social differences in the utilization of medical services by children and adolescents in Germany. Results of the cross-sectional KiGGS Wave 2 study

**DOI:** 10.17886/RKI-GBE-2018-098

**Published:** 2018-12-12

**Authors:** Thomas Lampert, Franziska Prütz, Alexander Rommel, Benjamin Kuntz

**Affiliations:** Robert Koch Institute, Berlin, Department of Epidemiology and Health Monitoring

**Keywords:** SOCIOECONOMIC STATUS, HEALTH DISPARITIES, HEALTH CARE, HEALTH MONITORING, KIGGS

## Abstract

Data from the German Health Interview and Examination Survey for Children and Adolescents (KiGGS) enable conclusions about the utilization of outpatient and inpatient medical services by children and adolescents accounting the family’s socioeconomic status (SES). Results from the second follow-up to the KiGGS survey (KiGGS Wave 2), which covers the years 2014 to 2017, clearly demonstrate that children and adolescents from families with a low SES visit specialists in general medicine, gynaecologists and psychiatrists, as well as child and adolescent psychiatrists, psychotherapists and psychologists more frequently. In contrast, children and adolescents from families with a high SES visit paediatric, dermatological, dental and orthodontic practices more often. No statistically significant differences between the status groups with regard to the utilization of outpatient medical services in hospitals were identified. However, children and adolescents from the low status group received inpatient hospital treatment more frequently and, on average, spent more nights in hospital. These results reflect status-specific differences both in disease prevalence and care, as well as in patterns related to the utilization of medical services.

## 1. Introduction

The German Health Interview and Examination Survey for Children and Adolescents (KiGGS) initially took place between 2003 and 2006. Since then, two follow-up studies have been conducted: the first took place between 2009 and 2012, and the second between 2014 and 2017. Importantly, the study’s results show that most children and adolescents in Germany grow up healthily. However, they also indicate that children and adolescents from socially disadvantaged families are far more likely to be affected by health problems than their peers from families with a better socioeconomic status [1-4]. The results from KiGGS confirm and complement the findings of other studies and surveys, such as the school entry health examinations by German public health service (Öffentlicher Gesundheitsdienst) and the Health Behaviour in School-aged Children (HBSC) study [5-7].

Social differences that are often linked to a family’s socioeconomic status (SES), in other words parental education, occupational status, and household income, are less pronounced when it comes to physical health. Very few, if any, identifiable differences exist within the distribution of many acute diseases such as influenza, conjunctivitis or diarrhoeal disease, and typical childhood diseases including rubella, chickenpox and scarlet fever [8]. The same is true of many childhood chronic diseases. In fact, children and adolescents from families with a higher SES are often more frequently affected by certain allergic diseases, such as atopic dermatitis [9]. However, the poorer health prospects of children and adolescents from socially disadvantaged families are more evident with regard to mental and psychosocial health. The results from the KiGGS study demonstrate that children and adolescents from families with a low SES have a significantly higher risk of mental health problems and psychological disorders such as depression, eating disorders and attention deficit/hyperactivity disorder (ADHD) as well as more frequent impairments to subjective well-being and health-related quality of life [4, 10]. In addition, the school entry health examinations show that impaired cognitive, emotional, linguistic and psychomotor development, among other conditions, are identified more frequently among socially disadvantaged children, and that these conditions can sometimes lead to a postponement of school enrolment [11-13].

**KiGGS Wave 2**

Second follow-up to the German Health Interview and Examination Survey for Children and Adolescents

**Data owner:** Robert Koch Institute

**Aim:** Providing reliable information on health status, health-related behaviour, living conditions, protective and risk factors, and health care among children, adolescents and young adults living in Germany, with the possibility of trend and longitudinal analyses

**Study design**: Combined cross-sectional and cohort study

**Cross-sectional study in KiGGS Wave 2**

**Age range:** 0-17 years

**Population:** Children and adolescents with permanent residence in Germany

**Sampling:** Samples from official residency registries - randomly selected children and adolescents from the 167 cities and municipalities covered by the KiGGS baseline study

**Sample size:** 15,023 participants

**KiGGS cohort study in KiGGS Wave 2**

**Age range:** 10-31 years

**Sampling:** Re-invitation of everyone who took part in the KiGGS baseline study and who was willing to participate in a follow-up

**Sample size:** 10,853 participants

**KiGGS survey waves**

► KiGGS baseline study (2003-2006), examination and interview survey
► KiGGS Wave 1 (2009-2012), interview survey
► KiGGS Wave 2 (2014-2017), examination and interview survey

More information is available at www.kiggs-studie.de/english

Significant social differences have also been identified regarding health behaviour. In accordance with the findings of the HBSC study, the results of the KiGGS study show that children and adolescents from families with a low SES follow less healthy diets. For example, they eat fresh fruit and vegetables less frequently every day and consume sugary soft drinks more frequently than their peers. In addition, children and adolescents from socially disadvantaged families are less physically active during their leisure time; this is particularly evident when it comes to their participation in sport. Accordingly, more children and adolescents from families with a low SES are overweight or even obese [14]. Moreover, adolescents from families with a low SES smoke more often than their peers, although the general decline in smoking in recent years is also evident among these children [15, 16].

So far, there are only few studies on social differences in health care. In this context it is important to distinguish between access to care, the utilization of services and the quality of care provision [17]. Whereas quality is usually addressed by disease-specific studies, aspects of access and utilization can be examined using population-wide surveys. However, only very few such studies on the utilization of health services by children and adolescents have been conducted. Nevertheless, the utilization of preventive health-care measures has been relatively well researched. For example, results from school entry health examinations indicate that participation in early detection examinations (called ‘U’ examinations in Germany) has increased in recent years. Participation rates among the low status group, however, still lag behind those of the medium and high status group [18, 19]. Social differences have also been identified in the uptake of important vaccinations, with the highest rates of vaccination often occurring in the medium status group against measles, mumps and rubella and human papillomavirus (HPV) [20, 21]. Recent results on the utilization of early detection examinations by children and on HPV vaccination of girls can be found in this issue of the Journal of Health Monitoring.

In contrast, only few studies put the focus on the influence of social differences on the utilization of outpatient and inpatient medical services by children and adolescents [22-24]. For example, data from the KiGGS baseline study showed that children and adolescents from families with low SES consult specialists in general medicine more frequently, but visit specialists such as ophthalmologists and dermatologists less often. The survey also demonstrated that these children more frequently receive in-patient treatment, and, when they do, tend to remain in hospital for a longer time [22]. Other surveys have focused on trends in utilization [23] or have concentrated on the utilization of specialist medical fields such as gynaecology [24], ophthalmology [25] and psychotherapy [26].

Against this background, this article investigates the extent to which social differences are currently reflected in the utilization of outpatient and inpatient medical services by children and adolescents in Germany. Data from KiGGS Wave 2, collected between 2014 and 2017, are used for this analysis. The discussion places the results in the context of existing research, sets out the findings of the KiGGS baseline study and KiGGS Wave 1, and highlights the changes that have occurred over the last ten years. Furthermore, this article complements two other articles that have already been published in the Journal of Health Monitoring that discuss the results gained from KiGGS Wave 2. These articles focus on socioeconomic differences in health behaviour and on social inequalities in health of children and adolescents [3, 4]. In addition, the results of further analyses on the utilization of paediatric and general medical services are described in a Fact sheet published in this issue of the Journal.

## 2. Methodology

### 2.1 Study design and sample

KiGGS is part of the health monitoring system at the Robert Koch Institute and includes repeated cross-sectional surveys of children and adolescents aged 0 to 17 (KiGGS cross-sectional study) that are representative for Germany. The KiGGS baseline study (2003-2006) was conducted as an examination and interview survey, the first follow-up study (KiGGS Wave 1, 2009-2012) as a telephone-based interview survey and KiGGS Wave 2 (2014-2017) as an examination and interview survey. However, in contrast to the KiGGS baseline study, one section of the participants was only interviewed and the other was examined in addition to being interviewed. A detailed description of the methodology used in KiGGS Wave 2 can be found elsewhere [27-30]. A total of 15,023 children and adolescents (7,538 girls, 7,485 boys) participated in the cross-sectional component of KiGGS Wave 2 (response rate 40.1%) [28]. The examination component involved 3,567 children and adolescents (1,801 girls, 1,766 boys; response rate 41.5%).

### 2.2 Indicators

The data on the utilization of outpatient and inpatient medical services used in the analyses were collected using a self-administered questionnaire. Parents filled out the questionnaires on behalf of children aged 13 or under, 14- to 17-year-olds completed the questionnaire themselves. The questionnaire included the following question on the utilization of outpatient medical services: ‘Please tell us which doctors in private practice of the following disciplines you have consulted for your child in the last 12 months and how often’. This, and the following questions, was adapted for use with the older children. The participants were expected to include home visits, but not hospital or rehabilitation centre visits. The participants were provided with a list of specialist doctors (formatted as a table in the questionnaire) that included the following groups: ‘paediatrician and general practitioner; specialists for internal medicine (such as cardiologists, pulmonologists and diabetologists); gynaecologists; ophthalmologists; orthopaedists; ear, nose and throat specialists; neurologists; psychiatrists, child and adolescent psychiatrists, medical psychotherapists; psychologists, psychological psychotherapists; surgeons; dermatologists; radiographers and radiologists; urologists; dentists and orthodontists’. The questionnaire began by asking which if any of these doctors had been visited during the last year. This was followed by a question aimed at gaining an exact indication of the number of times that each group of doctors had been visited during the last year. It could also be noted in the questionnaire that another physician not included in the aforementioned groups of physicians had been consulted, or that the child or adolescent had not consulted a doctor at all during the past year.

In addition to the utilization of services provided in doctor’s surgeries, the questionnaire focused on the utilization of outpatient medical services in hospitals. As such, the parents were asked, ‘Has your child received outpatient treatment in an outpatient clinic, first aid unit or medical centre affiliated to a hospital’s outpatient department during the last 12 months?’ This question could be answered ‘yes’ or ‘no’. Only treatment that did not involve an overnight stay was to be included. In addition, the survey collected data about how frequently children visited such facilities during the last year.

Regarding the utilization of inpatient medical services, the parents were asked: ‘Has your child been admitted to hospital (and been kept in overnight) during the last 12 months?’ This question could also be answered ‘yes’ or ‘no’. Where applicable, the number of nights the child spent in hospital was also to be provided.

Social differences in the utilization of outpatient and inpatient medical services were assessed using the family’s socioeconomic status (SES). Data on SES were collected for KiGGS Wave 2 using an index based on the information provided by parents about their education, occupational status and income. This was weighted according to the number and age of the people living in a respective household (net equivalised income) [31]. The operationalisation applied in KiGGS Wave 2 largely corresponds to the procedure introduced in KiGGS Wave 1 [42]. Three groups – a low, medium and high status group – were used for the analyses. The low and high status groups accounted for around 20% each, and the medium status group for 60% of the study population [31]. Details about the way in which SES was measured can be found in a methodological paper published in issue 1/2018 of the Journal of Health Monitoring.

### 2.3 Statistical analysis

The analyses are based on data from 14,468 participants (7,298 girls, 7,170 boys) aged between 0 and 17. Depending on the indicator used, various numbers of participants had to be excluded from the analyses as certain information was missing. The results are stratified by gender and SES using prevalences (frequencies) with 95 % confidence intervals (95 % CI). In addition, adjusted odds ratios (aOR) with 95% confidence intervals, determined on the basis of logistic regression analyses, are presented.

They indicate the factor by which the statistical probability is increased for a particular health outcome to be present in the low or medium status groups compared to the high status group, which was defined as the reference category. The varying compositions of the status groups were statistically controlled for in terms of age, gender and migration background [33] in order to prevent these factors from leading to biased results.

The calculations were carried out using a weighting factor that corrects deviations within the sample from the population structure with regard to regional structure (rural area/urban area), age (in years), gender, federal state (as at 31 December 2015), German citizenship (as at 31 December 2014) and the parents’ level of education according to the Comparative Analysis of Social Mobility in Industrial Nations (CASMIN) system [34] (microcensus 2013 [35]).

All analyses were performed using Stata 14.2 (Stata Corp., College Station, TX, USA, 2015) and the KiGGS Wave 2 data set (version 9). Stata survey commands were used during all of the analyses to account for any clustering that might have occurred due to the selection of the sample points and the weighting applied to calculate confidence intervals and p-values [36]. A statistically significant difference between groups was assumed to have been demonstrated when p-values were lower than 0.05.

## 3. Results

According to data from KiGGS Wave 2, paediatricians are among the groups of doctors most often visited by children and adolescents on an outpatient basis (Table 1). At 72.7%, nearly three-quarters of parents stated that they had taken their 0- to 17-year-old children to see a paediatrician during the last 12 months. A similarly high prevalence of 74.2% is reported only for the group of dentists and orthodontists. Ophthalmologists (29.2%), specialists in general medicine (25.2%), specialists in ear, nose and throat medicine (17.0%), orthopaedists (14.0%) and dermatologists (11.9%) were also visited relatively frequently. The prevalences for all of the other groups of doctors were between one and seven per cent (Table 1). Differences between girls and boys were only identified in the utilization of ear, nose and throat specialists and surgeons, with both groups of doctors visited more often by boys than girls (p<0.01 each).

It should also be noted that specialists in general medicine are more frequently visited by children and adolescents with a low or medium socioeconomic status than by those with a high SES (p<0,001; Table 2 and Annex Table 1). In terms of gynaecology (p<0.001) and psychiatry (p<0.001), and psychological psychotherapy (p<0.01), utilization was higher in the low status group than in the high status group. With regard to gynaecology and psychotherapy, however, utilization was higher among the medium status group compared to the high status group. In contrast, other groups of doctors are more frequently visited by children and adolescents from families with a high SES. This applies for paediatricians (p<0.001), dermatologists (p<0.05) and dentists and orthodontists (p<0.001), in the latter, however, especially in comparison with the high to the low, but not to the medium status group.

The results of the multivariate analyses, which are controlled for age, migration background and, in the overall models, also for gender, confirm the descriptive results (Table 3 und Annex Table 2). The analyses demonstrate that the utilization of general medical services among the low status group is 1.4 times (p<0.01) higher and among the medium status group 1.5 times (p<0.001) higher than among members of the high status group. Gynaecologists are visited by the low status group 1.6 times more often (p<0.05), and psychiatrists (p<0.001) and psychotherapists (p<0.001) more than twice as often. With regard to the utilization of paediatric (p<0.05), dermatological (p<0.001) and dental or orthodontic practices (p<0.001), the adjusted odds ratios presented indicate a 1.3 to 1.6 times increased utilization in the high compared to the low status group.

In addition, data from KiGGS Wave 2 show that 23.3% of children and adolescents aged between 0 and 17 have received outpatient medical services in hospitals during the past 12 months. This includes care provided in outpatient clinics, first aid units and medical centres. The utilization among girls and boys was very similar, at 22.4% and 24.2%, respectively. In addition, no significant differences between the status groups were identified (Table 4). On average, the children and adolescents who received outpatient medical services in hospitals during the last 12 months visited the out-patient centres on average 1.7 times. Again, no significant differences were identified in terms of gender or socioeconomic status.

Finally, 8.4% of children and adolescents aged between 0 and 17 received inpatient medical services at least once during the last 12 months and spent at least one night in hospital (Table 4). No significant differences between the genders were identified (girls 8.3%, boys 8.5%). On average, the children and adolescents who received inpatient medical services during the past 12 months spent about seven nights in hospital. Girls tended to remain longer in hospital than boys (eight versus five nights). And a slightly higher proportion of children and adolescents from the low status group received inpatient medical services within one year (8.4%) compared to their peers from the high status group (6.8%). Importantly, even after differences in the composition of the status groups (age, migration background and gender) had been controlled for (Table 4), the risk of having spent at least one night in hospital within the last 12 months is 1.4 times higher in both the low and medium status groups than in the reference group of children and adolescents with high SES. In addition, children and adolescents from the low status group spent more than twice as many nights in hospital (on average around ten nights) as their peers from the high status group (four nights). The gender-differentiated analysis shows that girls from families with a low SES received inpatient medical services more frequently and on average for a longer period during the last 12 months than girls from families with high SES. No significant differences were identified among boys in this respect.

## 4. Discussion

The data from KiGGS Wave 2 indicate that there are social differences in the utilization of outpatient and inpatient medical services. With regard to outpatient medical services, children and adolescents from families with a low SES are more likely to visit specialists in general medicine, gynaecologists, psychiatrists and psychotherapists. Practices for paediatrics and dermatology as well as dental and orthodontic practices, on the other hand, are more frequently visited by children and adolescents from families with high SES. No significant differences were identified between the status groups in relation to the utilization of outpatient medical services in orthopaedics, ophthalmology, surgery, ear, nose and throat medicine, neurology, urology or radiology or in outpatient medical services in hospitals. This also includes health care provided in outpatient clinics, first aid units or medical centres in hospitals. However, a slightly higher proportion of children and adolescents with a low SES received inpatient medical services during the past 12 months. In addition, of the children and adolescents who received inpatient medical services, those from families with low SES spent on average more nights in hospital.

Similar results were identified from the data collected for the KiGGS baseline study, which was carried out between 2003 and 2006. The data shows that the low status group had a more frequent utilization of services provided by specialists in general medicine. Moreover, it also shows that children and adolescents from the high status group are more likely to visit paediatricians and dermatologists. However, the social differences in the utilization of ophthalmologists seen in the KiGGS baseline study no longer exist in KiGGS Wave 2.

A further difference between the two survey waves can be seen in the utilization of gynaecology: although no significant status-specific differences were identified in the data collected for the KiGGS baseline study [22], KiGGS Wave 2 found that girls with a low or medium SES visited gynaecologists more frequently. The underlying trend towards this change was already clear from the data collected for KiGGS Wave 1. KiGGS Wave 1 found a significant increase in the utilization of services provided by gynaecologists among girls aged between 14 and 17 in the low and medium status group during the 2009 to 2012 survey period compared to the KiGGS baseline study. In contrast, no change in utilization was identified among girls from the high status group [24]. However, the different levels of utilization of gynaecological services both in terms of socioeconomic status and over time can be related to various other factors. For example, the shift towards earlier menstruation could play a role. These changes could mean that girls now have a greater need for advice from gynaecologists at an earlier age (due to issues relating to menstruation) [24]. In addition, gynaecologists are also providing new services, such as vaccination against HPV and chlamydia screening [24]. However, the changes in the utilization of gynaecological services over time and the reasons for them can only be explained using age-differentiated analyses.

When interpreting the results, it is important to note that social differences in the utilization of medical services may reflect differences in the prevalence and severity of illnesses and health complaints (and therefore in care needs). Thus, the more frequent use of psychiatric and psychotherapeutic care among children and adolescents from families with low SES should be seen against the background of the significantly increased risk of mental health problems and disorders compared to peers with higher SES [4, 37]. More frequent stays in hospitals, and, in particular, the greater number of nights that they remain in hospital, could indicate that children and adolescents with a low SES face more serious diseases and worse unintentional injuries. Analyses of the KiGGS baseline study data show that children from families with a low SES undergo operations more frequently [22]. This could be due to different medical needs as well as the fact that social factors, medical opinions and the availability of care also play a role in this situation, particularly when it comes to preference sensitive operations such as tonsillectomies (the surgical removal of the palatine tonsils) [22].

Although differences in utilization between the status groups can be explained by the choices made by the patients themselves, they can also be caused by barriers to care. Other studies have shown that adults in the low status group are more likely to turn to general practitioners when they have health problems, whereas people in the high status groups tend to visit specialists of other medical disciplines directly [38, 39]. As people with a low SES are more likely to choose a general practitioner as their first point of contact, they may only gain specialist care through referral [40]. Moreover, children are affected by their parents’ attitudes and views, and two groups of specialists are available to provide basic medical care to children: specialists in paediatrics and general medicine. Alongside social differences, other factors such as urban/rural differences play an important role [22, 23, 41, 42]. As such, further context analyses are needed to supplement the analyses presented here.

Although the KiGGS data offer a comparatively good basis with which to analyse social differences in the utilization of outpatient and inpatient medical services, they also face a number of limitations. For example, it remains unclear to what extent children and adolescents with a high medical need are adequately reached by population-based studies. A certain level of bias due to their non-participation, therefore, cannot be excluded. In addition, KiGGS Wave 2 does not provide any information about the reasons why a particular person sought medical treatment or advice. Furthermore, no conclusions can be made about the reasons that led to the utilization of outpatient medical services in a hospital or for inpatient stay. Accordingly, no conclusions can be drawn about the extent to which supply actually reflects demand.

However, this analysis provides important information about the social differences that currently exist in the utilization of outpatient and inpatient medical services by children and adolescents. In this respect, the data on utilization represents the actual use (realised access) of health services under the given conditions, such as barriers to access or individual preferences. Therefore, it does not merely reflect the supply needs of the population. Moreover, it can be assumed that the pronounced social differences identified in utilization and which occur regardless of medical need, pose a quality problem for healthcare systems (in terms of inequitable access) [43]. In order to address the issue of more or less adequate levels of utilization and to provide an assessment of social differences, more specific analyses are needed focusing on the utilization of particular health care services due to the presence of a specific disease [44].

## Key statements

- Children and adolescents from families with a low socioeconomic status visit general practitioners, gynaecologists, psychiatrists and psychotherapists more frequently.
- Children and adolescents from families with a high socioeconomic status visit paediatric, dermatological as well as dental and orthodontic practices more frequently.
- A slightly higher proportion of children from families with a low socioeconomic status received inpatient hospital treatment within a given year.
- On average, of all the children who received inpatient hospital treatment, children from families with a low socioeconomic status tended to spend more nights in hospital.

## Figures and Tables

**Table 1:** Utilization of physicians and dentists in private practice in the last 12 months by 0- to 17-year-olds according to gender and medical discipline (n=7,298 girls, n=7,170 boys)* Source: KiGGS Wave 2 (2014-2017)

Girls			Boys		Total	
%		(95% CI)	%	(95% CI)	%	(95% CI)
Paediatrics	72.8	(70.8-74.7)	72.7	(70.9-74.4)	72.7	(71.0-74.4)
General medicine	25.9	(23.5-28.5)	24.6	(22.2-27.1)	25.2	(23.0-27.6)
Internal medicine (e.g. cardiology, pneumology and diabetology)	4.7	(4.1-5.3)	4.2	(3.5-4.9)	4.4	(4.0-4.9)
Gynaecology	10.9	(10.1-11.9)		–		–
Ophthalmology	31.3	(29.5-33.1)	27.2	(25.6-28.8)	29.2	(27.9-30.5)
Orthopaedics	15.0	(13.9-16.2)	13.1	(12.1-14.2)	14.0	(13.1-14.9)
Ear, nose and throat medicine	15.6	(14.4-17.0)	18.3	(17.1-19.7)	17.0	(16.0-18.1)
Neurology	1.2	(0.9-1.6)	1.2	(0.9-1.6)	1.2	(1.0-1.5)
Psychiatry, child and adolescent psychiatry (incl. medical psychotherapists)	3.6	(3.0-4.4)	3.9	(3.4-4.5)	3.8	(3.4-4.3)
Psychotherapy (incl. psychological psychotherapists)	2.6	(2.1-3.1)	2.4	(2.0-2.8)	2.5	(2.2-2.8)
Surgery	5.2	(4.5-5.9)	6.9	(6.0-7.9)	6.0	(5.5-6.7)
Dermatology	12.3	(11.3-13.3)	11.6	(10.5-12.7)	11.9	(11.1-12.7)
Radiology	6.5	(5.8-7.4)	6.3	(5.6-7.1)	6.4	(5.8-7.0)
Urology		–	2.9	(2.3-3.6)		–
Dentistry, Orthodontics	75.4	(74.2-76.6)	73.0	(71.5-74.5)	74.2	(73.1-75.3)

* Number of cases depending on considered outcome

CI=confidence interval

**Table 2:** Social differences in the utilization of physicians and dentists in private practice in the past 12 months by 0- to 17-year-olds according to socioeconomic status and medical discipline (n=7,261 girls, n=7,129 boys)* Source: KiGGS Wave 2 (2014-2017)

Socioeconomic status						
Low			Medium		High	
%		(95% CI)	%	(95% CI)	%	(95% CI)
Paediatrics	70.4	(67.0-73.5)	72.0	(70.0-73.9)	77.7	(75.6-79.6)
General medicine	24.7	(22.0-27.7)	27.3	(24.8-30.0)	19.1	(16.6-21.8)
Internal medicine (such as cardiology, pneumology and diabetology)	3.1	(2.3-4.3)	4.8	(4.2-5.4)	4.5	(3.7-5.4)
Gynaecology^1^	13.9	(11.2-17.0)	11.2	(10.2-12.3)	7.0	(5.7-8.6)
Ophthalmology	27.6	(25.1-30.3)	29.6	(28.0-31.2)	29.5	(27.4-31.6)
Orthopaedics	11.7	(10.1-13.6)	14.8	(13.7-16.1)	13.6	(12.2-15.2)
Ear, nose and throat medicine	17.7	(15.3-20.4)	17.1	(15.9-18.3)	15.9	(14.4-17.4)
Neurology	1.5	(0.9-2.6)	1.3	(1.0-1.6)	0.8	(0.5-1.2)
Psychiatry, child and adolescent psychiatry (incl. medical psychotherapists)	5.3	(4.1-6.8)	3.6	(3.2-4.2)	2.6	(2.1-3.4)
Psychotherapy (incl. psychological psychotherapists)	3.4	(2.4-4.8)	2.4	(2.0-2.9)	1.5	(1.1-1.9)
Surgery	5.3	(4.1-6.8)	6.4	(5.7-7.2)	5.6	(4.6-6.7)
Dermatology	9.9	(8.2-11.9)	12.0	(11.1-13.0)	13.4	(12.1-14.8)
Radiology	6.2	(5.0-7.7)	6.7	(5.9-7.5)	5.9	(5.1-6.8)
Urology^2^	1.8	(0.9 – 3.3)	3.6	(2.8-4.6)	2.0	(1.5-2.9)
Dentistry, Orthodontics	66.2	(63.2-69.1)	76.6	(75.2-77.9)	75.1	(73.1-77.1)

*Number of cases depending on considered outcome

^1^ Utilization refers only to girls

^2^ Utilization refers only to boys

CI=confidence interval

**Table 3:** Social differences in the utilization of physicians and dentists in private practice in the past 12 months by 0- to 17-year-olds according to socioeconomic status and medical discipline. Results of logistic regressions controlled for age, gender and migration background (n=7,261 girls, n=7,129 boys)* Source: KiGGS Wave 2 (2014-2017)

Socioeconomic status low versus high			Socioeconomic status medium versus high	
aOR		(95% CI)	aOR	(95% CI)
Paediatrics	**0.78**	**(0.62-0.96)**	**0.84**	**(0.73-0.96)**
General medicine	**1.41**	**(1.15-1.73)**	**1.52**	**(1.30-1.78)**
Internal medicine (such as cardiology, pneumology and diabetology)	0.67	(0.45-1.00)	1.04	(0.83-1.31)
Gynaecology^1^	**1.62**	**(1.11-2.35)**	**1.36**	**(1.04-1.77)**
Ophthalmology	0.95	(0.81-1.11)	0.98	(0.87-1.10)
Orthopaedics	0.82	(0.66-1.03)	1.05	(0.90-1.23)
Ear, nose and throat medicine	1.18	(0.96-1.46)	1.11	(0.97-1.28)
Neurology	1.98	(0.95-4.14)	1.64	(0.97-2.77)
Psychiatry, child and adolescent psychiatry (incl. medical psychotherapists)	**2.45**	**(1.64-3.66)**	1.33	(0.99-1.78)
Psychotherapy (incl. psychological psychotherapists)	**2.22**	**(1.37-3.60)**	**1.57**	**(1.10-2.22)**
Surgery	1.06	(0.76-1.49)	1.16	(0.91-1.48)
Dermatology	**0.61**	**(0.49-0.76)**	**0.81**	**(0.70-0.93)**
Radiology	1.06	(0.81-1.39)	1.05	(0.84-1.31)
Urology^2^	0.87	(0.45-1.70)	**1.78**	**(1.11-2.84)**
Dentistry, Orthodontics	**0.63**	**(0.52-0.76)**	1.01	(0.88-1.15)

* Number of cases depending on considered outcome

^1^ Utilization refers only to girls

^2^ Utilization refers only to boys

aOR=adjusted odds ratio, CI=confidence interval, bold=statistically significant (p<0.05)

**Table 4:** Utilization of outpatient and inpatient medical services in hospitals over the last 12 months by 0- to 17-year-olds according to gender and socioeconomic status (n=7,276 girls, n=7,176 boys)* Source: KiGGS Wave 2 (2014-2017)

Utilization of outpatient medical services in hospitals					Number of visits^1^	Utilization of inpatient medical services in hospitals				Number of nights spent in hospitals (in days)^2^
%		(95% CI)	aOR	(95% CI)	Mean value	%	(95% CI)	aOR	(95% CI)	Mean value
**Girls**	22.4	(20.9-24.0)		–	1.6	8.3	(7.7-9.4)		–	8.1
**Socioeconomic status**										
Low	22.2	(18.6-26.2)	1.24	(0.95-1.61)	1.8	10.7	(8.1-14.0)	**1.93**	**(1.39-2.67)**	12.7
Medium	23.0	(21.4-24.7)	**1.18**	**(1.01-1.39)**	1.6	8.0	(7.0-9.1)	1.25	(0.97-1.62)	7.3
High	20.8	(18.5-23.4)		Ref.	1.5	6.8	(5.5-8.4)		Ref.	4.7
**Boys**	24.2	(22.8-25.7)		–	1.7	8.5	(7.7-9.4)		–	5.1
**Socioeconomic status**										
Low	20.8	(17.6-24.4)	0.85	(0.65-1.11)	1.9	6.3	(4.5-8.7)	1.02	(0.65-1.61)	5.7
Medium	24.8	(23.0-26.7)	0.99	(0.84-1.17)	1.7	9.8	(8.5-11.1)	**1.56**	**(1.18-2.06)**	5.1
High	25.9	(23.5-28.4)		Ref.	1.6	6.9	(5.6-8.3)		Ref.	4.2
**Total**	23.3	(22.2-24.5)		–	1.7	8.4	(7.8-9.1)		–	6.6
**Socioeconomic status**										
Low	21.5	(19.1-24.0)	1.01	(0.85-1.21)	1.9	8.4	(6.9-10.2)	**1.43**	**(1.13-1.82)**	9.9
Medium	23.9	(22.7-25.2)	1.07	(0.96-1.20)	1.7	8.9	(8.1-9.7)	**1.40**	**(1.18-1.66)**	6.1
High	23.5	(21.8-25.2)		Ref.	1.5	6.8	(6.0-7.8)		Ref.	4.5

* Number of cases depending on considered outcome

^1^ Average number of visits for participants who have used outpatient hospital services in the last 12 months

^2^ Average number of nights spent in hospitals for participants who have been hospitalised in the last 12 months

aOR=adjusted odds ratio, CI=confidence interval, Ref.=Reference, bold=statistically significant (p <0.05)

## Annex Table 1 Social differences in the utilization of physicians and dentists in private practice in the past 12 months by 0- to 17-year-olds according to gender, socioeconomic status and medical discipline (n=7.261 girls, n=7.129 boys)* Source: KiGGS Wave 2 (2014-2017)

**Table:**

Girls							Boys					
Socioeconomic status							Socioeconomic status					
Low			Medium		High		Low		Medium		High	
%		(95% CI)	%	(95% CI)	%	(95% Cl)	%	(95%-KI)	%	(95% CI)	%	(95% CI)
Paediatrics	70.3	(65.7-74.6)	72.1	(69.8-74.3)	77.4	(74.5-80.0)	70.4	(65.8-74.7)	72.0	(69.6-74.2)	77.9	(75.5-80.1)
General medicine	26.2	(22.6-30.2)	27.9	(25.0-31.0)	19.4	(16.4-22.8)	23.3	(19.8-27.3)	26.7	(23.9-29.8)	18.8	(16.0-21.9)
Internal medicine (such as cardiology, pneumology and diabetology)	3.6	(2.3-5.6)	5.1	(4.3-6.0)	4.3	(3.3-5.5)	2.7	(1.6-4.4)	4.5	(3.7-5.4)	4.7	(3.6-6.2)
Gynaecology	13.9	(11.2-17.0)	11.2	(10.2-12.3)	7.0	(5.7-8.6)		–		–		–
Ophthalmology	30.8	(26.6-35.4)	31.3	(29.2-33.5)	31.4	(28.6-34.4)	24.8	(21.5-28.4)	27.8	(25.8-29.9)	27.6	(25.0-30.4)
Orthopaedics	13.6	(10.9-16.8)	15.7	(14.2-17.3)	14.0	(11.9-16.3)	10.0	(7.9-12.7)	13.9	(12.6-15.4)	13.4	(11.6-15.3)
Ear, nose and throat medicine	15.9	(12.7-19.8)	16.3	(14.9-17.8)	13.2	(11.4-15.3)	19.3	(16.1-23.0)	17.9	(16.3-19.6)	18.3	(16.3-20.5)
Neurology	2.3	(1.2-4.4)	0.9	(0.6-1.3)	0.9	(0.5-1.7)	0.8	(0.4-1.6)	1.6	(1.1-2.2)	0.6	(0.3-1.2)
Psychiatry, child and adolescent psychiatry (incl. medical psychotherapists)	5.8	(4.2-8.1)	3.5	(2.8-4.4)	1.9	(1.3-2.8)	4.8	(3.3-6.9)	3.8	(3.1-4.5)	3.3	(2.6-4.3)
Psychotherapy (incl. psychological psychotherapists)	4.3	(3.0-6.3)	2.4	(1.9-3.0)	1.1	(0.7-1.7)	2.6	(1.5-4.5)	2.5	(2.0-3.1)	1.8	(1.3-2.6)
Surgery	6.1	(4.3-8.4)	5.1	(4.3-5.9)	4.4	(3.3-5.9)	4.6	(3.1-6.9)	7.7	(6.6-9.0)	6.6	(5.2-8.4)
Dermatology	10.6	(8.3-13.4)	12.4	(11.3-13.7)	13.2	(11.5-15.2)	9.3	(7.0-12.1)	11.6	(10.3-12.9)	13.6	(11.8-15.6)
Radiology	7.6	(5.5-10.3)	6.4	(5.5-7.4)	6.0	(4.9-7.4)	5.0	(3.7-6.8)	6.9	(5.9-8.2)	5.7	(4.7-7.0)
Urology		–		–		–	1.8	(0.9-3.3)	3.6	(2.8-4.6)	2.0	(1.5-2.9)
Dentistry, Orthodontics	69.1	(64.9-72.9)	77.2	(75.5-78.8)	76.4	(73.6-78.9)	63.7	(59.2-67.9)	76.0	(74.1-77.8)	74.0	(71.1-76.7)

*Number of cases depending on considered outcome

CI=confidence interval

## Annex Table 2 Social differences in the utilization of physicians and dentists in private practice in the past 12 months by 0- to 17-year-olds according to gender, socioeconomic status and medical discipline. Results of logistic regressions controlled for age and migration background (n=7,261 girls, n=7,129 boys)* Source: KiGGS Wave 2 (2014-2017)

**Table:**

Girls					Boys			
Socioeconomic status low versus high			Socioeconomic status medium versus high		Socioeconomic status low versus high		Socioeconomic status medium versus high	
aOR		(95% CI)	aOR	(95% CI)	aOR	(95% CI)	aOR	(95% CI)
Paediatrics	0,87	(0,62 – 1,21)	0,89	(0,73 – 1,09)	**0,70**	**(0,54 – 0,91)**	**0,80**	**(0,68 – 0,95)**
General medicine	**1,35**	**(1,03 – 1,78)**	**1,49**	**(1,20 – 1,85)**	**1,45**	**(1,11 – 1,90)**	**1,55**	**(1,27 – 1,88)**
Internal medicine (such as cardiology, pneumology and diabetology)	0,75	(0,42 – 1,35)	1,15	(0,84 – 1,57)	0,60	(0,33 – 1,11)	0,96	(0,70 – 1,30)
Gynaecology^1^	**1,62**	**(1,11 – 2,35)**	**1,36**	**(1,04 – 1,77)**		–		–
Ophthalmology	0,93	(0,73 – 1,17)	0,95	(0,81 – 1,10)	0,95	(0,75 – 1,21)	1,01	(0,86 – 1,18)
Orthopaedics	0,89	(0,63 – 1,26)	1,09	(0,87 – 1,36)	0,76	(0,56 – 1,03)	1,02	(0,84 – 1,23)
Ear, nose and throat medicine	1,26	(0,91 – 1,73)	**1,29**	**(1,05 – 1,58)**	1,14	(0,87 – 1,48)	0,99	(0,83 – 1,18)
Neurology	2,17	(0,80 – 5,91)	0,96	(0,48 – 1,96)	1,56	(0,57 – 4,26)	**2,68**	**(1,26 – 5,69)**
Psychiatry, child and adolescent psychiatry (incl. medical psychotherapists)	**3,54**	**(2,01 – 6,23)**	**1,71**	**(1,09 – 2,70)**	**1,80**	**(1,08 – 3,01)**	1,12	(0,80 – 1,56)
Psychotherapy (incl. psychological psychotherapists)	**3,55**	**(1,81 – 6,97)**	**2,02**	**(1,17 – 3,49)**	1,37	(0,71 – 2,66)	1,30	(0,82 – 2,05)
Surgery	1,49	(0,92 – 2,40)	1,10	(0,76 – 1,58)	0,78	(0,47 – 1,30)	1,21	(0,88 – 1,67)
Dermatology	**0,63**	**(0,45 – 0,89)**	0,83	(0,68 – 1,01)	**0,57**	**(0,41 – 0,79)**	**0,78**	**(0,64 – 0,95)**
Radiology	1,21	(0,75 – 1,95)	0,96	(0,74 – 1,25)	0,91	(0,62 – 1,34)	1,15	(0,85 – 1,54)
Urology^2^		–		–	0,87	(0,45 – 1,70)	**1,78**	**(1,11 – 2,84)**
Dentistry, Orthodontics	**0,58**	**(0,45 – 0,75)**	0,92	(0,77 – 1,12)	**0,66**	**(0,50 – 0,87)**	1,08	(0,90 – 1,30)

* Number of cases depending on considered outcome

aOR = adjusted odds ratio, CI=confidence interval, bold=statistically significant (p<0.05)
